# A Versatile Hermetically Sealed Microelectronic Implant for Peripheral Nerve Stimulation Applications

**DOI:** 10.3389/fnins.2021.681021

**Published:** 2021-07-22

**Authors:** Dai Jiang, Fangqi Liu, Henry T. Lancashire, Timothy A. Perkins, Matthew Schormans, Anne Vanhoestenberghe, Nicholas De N. Donaldson, Andreas Demosthenous

**Affiliations:** ^1^Department of Electronic and Electrical Engineering, University College London, London, United Kingdom; ^2^Department of Medical Physics and Biomedical Engineering, University College London, London, United Kingdom; ^3^Division of Surgery and Interventional Science, Aspire Centre for Rehabilitation Engineering and Assistive Technology, University College London, London, United Kingdom

**Keywords:** hermetic seal package, implantable stimulator, microelectronics, power and data telemetry, wireless stimulation control

## Abstract

This article presents a versatile neurostimulation platform featuring a fully implantable multi-channel neural stimulator for chronic experimental studies with freely moving large animal models involving peripheral nerves. The implant is hermetically sealed in a ceramic enclosure and encapsulated in medical grade silicone rubber, and then underwent active tests at accelerated aging conditions at 100°C for 15 consecutive days. The stimulator microelectronics are implemented in a 0.6-μm CMOS technology, with a crosstalk reduction scheme to minimize cross-channel interference, and high-speed power and data telemetry for battery-less operation. A wearable transmitter equipped with a Bluetooth Low Energy radio link, and a custom graphical user interface provide real-time, remotely controlled stimulation. Three parallel stimulators provide independent stimulation on three channels, where each stimulator supports six stimulating sites and two return sites through multiplexing, hence the implant can facilitate stimulation at up to 36 different electrode pairs. The design of the electronics, method of hermetic packaging and electrical performance as well as *in vitro* testing with electrodes in saline are presented.

## Introduction

Direct interaction with neural pathways through active implantable devices has become an increasingly effective therapeutic approach for treating neurological disorders and organ defects, or replacing lost body function. Traditional clinical applications include cochlear implants for hearing loss, deep brain stimulation (DBS) for epilepsy and Parkinson’s disease, and pacemakers for heart defects. More recent research includes epidural spinal cord stimulation for restoring coordinated locomotion in lower limbs (Capogrosso et al., 2016; Formento et al., 2018), peripheral nerve stimulation for creating tactile sensation after amputation (Raspopovic et al., 2014; Zollo et al., 2019), and vagus nerve stimulation for regulating organ function through neuromodulation in order to reinstate a healthy balance (Famm et al., 2013; Pavlov and Tracey, 2017).

Research on implantable active neural interface devices require chronic studies in animal models in order to gain a thorough understanding of the mechanism of neural diseases and disorders. Implantable devices used in these studies require accurate and highly selective neural stimulation at multiple sites. The stimulation should be highly programmable to support closed-loop neural intervention. A variety of implantable stimulator designs has been reported in the literature in the past two decades. They can be divided into three major categories:

1) *Implants adapted from commercially available devices* (Capogrosso et al., 2016; Boutros et al., 2019): Although these implants have proven reliability, they are often limited by their inflexibility, coarse programmability, and low channel count;
2) *Implants without hermetic packaging* (Xu et al., 2015; Lee et al., 2018; Williams et al., 2020): In these implants the electronics are encapsulated in silicone rubber or epoxy. This is a widely adopted approach because of its simple process and low cost. These devices, however, are often used only in short-term animal studies due to the lack of adequate hermetic protection;
3) *In-house made prototype chronic implants:* Some of these devices are packaged in precious metals (Hart et al., 2011; Sun and Morrell, 2014; Zamora et al., 2020) and are expensive for production. Others are simple electronic circuits sealed in miniaturized glass packages (Loeb et al., 2001; Sivaji et al., 2019), where the channel count and programmability are limited.

This article presents the design, implementation, and evaluation of a versatile fully implantable multi-channel stimulator implant for chronic animal studies targeting the peripheral nervous system. The implant is hermetically packaged in a ceramic enclosure and is cost effective as a research platform. Inductive wireless powering removes the need for an implanted battery, avoiding potential battery failure. A bidirectional, high-speed communication channel facilitates real-time programming of the implant from a remote external host computer allowing free movement of the animal. Results from accelerated aging tests at 100°C for 15 consecutive days demonstrate that the implantable stimulator is suitable for chronic implantation.

The rest of the paper is organized as follows. Section “Materials and Methods” describes the design and fabrication of the hardware system, and the operation procedure for remote real-time stimulation control. Section “Results” show the electrical performance of the device as well as its suitability for chronic implantation. Section “Discussion and Conclusion” elaborates on the findings and provides concluding remarks and future directions.

## Materials and Methods

### System Architecture

**FIGURE 1:**
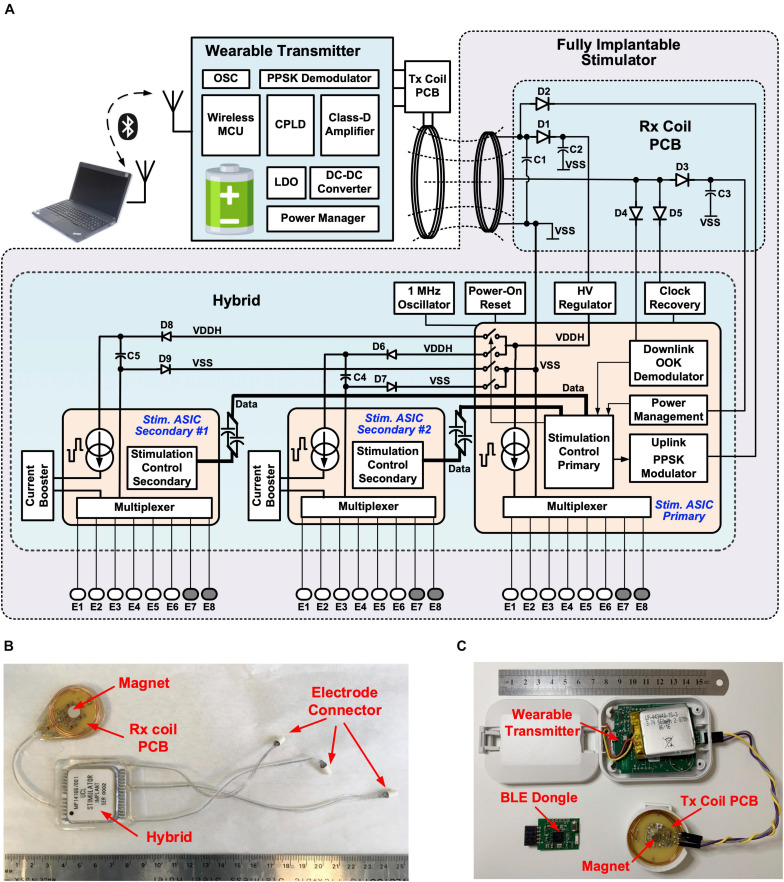
Wireless, fully implantable multi-channel stimulator: **(A)** System architecture; **(B)** Implantable stimulator; **(C)** Wearable transmitter and BLE dongle.

The wireless multi-channel stimulator system consists of a hermetically sealed, fully implantable stimulator, as shown in Figure 1C, and a wearable transmitter, as shown in Figure 1B. The implant does not contain a built-in energy source. Power is supplied by the wearable transmitter over a wireless power transfer link comprising two inductively coupled coils, which also provides bidirectional half-duplex communication between the transmitter and the implant. The operation of the transmitter is managed by a CC2640 microcontroller (MCU), which also provides a Bluetooth Low Energy (BLE) radio link. This allows the stimulation from the implant to be controlled from a remote host computer. A custom BLE dongle was designed and fabricated for the radio link of the host computer, as shown in Figure 1B. Dedicated software with a graphical user interface (GUI) has been developed for wirelessly controlling the operation of the implantable stimulator from the host computer. In experiments with free-moving animals, the wearable transmitter could be mounted onto the animal subject in a jacket or backpack, with the transmitter (Tx) coil in the inductive link aligned to the implanted receiver (Rx) coil. Researchers can set stimulation parameters on-the-fly from the GUI, where the setting commands are transmitted via an USB-UART interface on the BLE dongle, then over the BLE link to the wearable transmitter, where the commands are relayed to the implant via the inductive link. This arrangement allows real-time control of neurostimulation by the implantable stimulator without the need to attach a cable to the animal subject.

The architecture of the wireless multi-channel stimulator system is shown in Figure 1A. The wearable transmitter comprises a rechargeable battery, power management modules, a class-D driver for driving the inductive link, wireless communication modules and a MCU. The implantable stimulator has three parallel stimulators. The primary stimulator facilitates communication and manages the stimulation settings on all the three stimulators. It has a current pulse generator providing biphasic current pulses up to 1 mA, which are multiplexed to six stimulating electrodes and two return electrodes, supporting up to 12 different electrode configurations. The primary stimulator control unit has three parallel finite-state machines (FSMs) for managing the stimulation settings on each stimulator. The FSMs for the two secondary stimulators trigger the local secondary stimulation control units to operate the current pulse generators, where the current amplitude is amplified by a current booster to up to 3 mA. Each secondary stimulator also supports up to 12 electrode configurations. Therefore, the implant can provide three independent channels of parallel current stimulation for up to 36 different electrode pairs.

As shown in Figure 1B, the implantable stimulator comprises a hybrid unit, where the stimulation electronics are mounted and sealed inside a ceramic package, a Rx coil printed circuit board (PCB) with a solenoid coil and tuning capacitors for inductive coupling, and three miniature connectors for connecting electrode arrays. The Rx coil PCB also has a neodymium rare earth magnet (8 mm × 3 mm, Duratool) to aid alignment with the Tx coil PCB outside the body, which also has a magnet, as shown in Figure 1C. The optimal working distance between the Tx and Rx coils is ∼ 1 cm. The hybrid, Rx coil PCB and connectors are joined with Cooner wires. The length between the hybrid and the Rx coil PCB is ∼ 55 mm, and between the hybrid and the connectors is up to 180 mm. This arrangement allows the Rx coil PCB to be implanted close to the skin for strong coupling, whilst the hybrid can be implanted in a relatively deeper, surgically suitable location. In addition, the implantable electrode connectors provide the flexibility of having the electrode cable at any desired length required to reach the targeted nerve. All the units are encapsulated in medical grade silicone rubber. The hermetic package and silicone encapsulation ensure the suitability of the stimulator for chronic implantation.

### Stimulator Circuits

**FIGURE 2:**
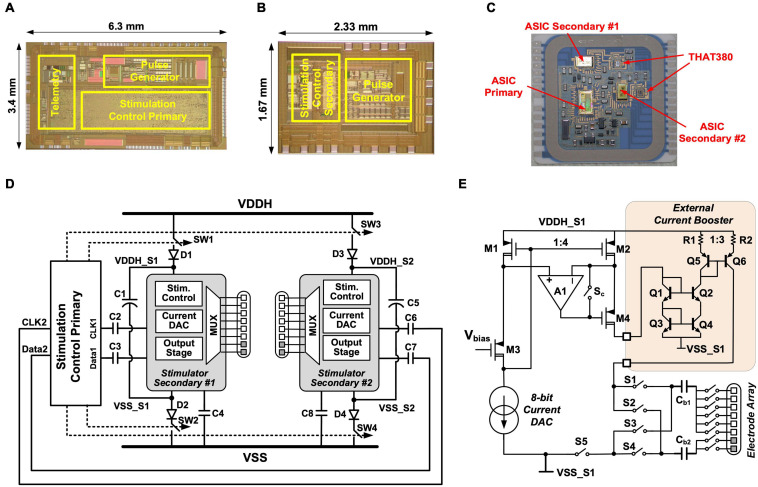
Stimulator circuits: **(A)** Microphotograph of the primary stimulator ASIC; **(B)** Microphotograph of the secondary stimulator ASIC; **(C)** Layout of the stimulator circuits inside the hybrid; **(D)** Schematic of the power isolation scheme for crosstalk reduction; **(E)** Integrated pulse generator on Stimulator Secondary #1 (the circuits for the integrated pulse generator on Stimulator Secondary #2 are identical).

The circuits of the three stimulators are integrated onto three application specific integrated circuits (ASICs) using a high-voltage (HV) 0.6 μm CMOS technology. Figures 2A,B show the microphotographs of the primary and secondary stimulator ASICs, respectively. Bare dies of the stimulator ASICs, as well as two commercially available THAT380 ICs, are wire-bonded directly to printed pads inside the hermetically sealed hybrid as shown in Figure 2C. In a conventional multi-channel stimulator design, channel interference is addressed by either interleaving the pulses from multiple channels (Zeng et al., 2008), or physically isolating the electrodes (Wong et al., 2007). The former approach correlates the channels hence reduces the degree of stimulation independence, while the latter approach increases the size of the electrode array, which may be limited by surgical constraints. To ensure truly multi-channel stimulation, the implant uses a novel power isolation scheme (Jiang et al., 2015). Figure 2D shows the circuit arrangement for this scheme. The two secondary stimulator ASICs are supplied from the 16 V supply rails VDDH and VSS through a switched connection, where the switches are controlled by the primary stimulation control logic. Before the onset of a biphasic pulse from a secondary stimulator, for example, Stimulator Secondary #1, the primary control logic sends the pulse amplitude setting to the secondary stimulation control logic via ac coupled data connections CLK1 and Data1, so that the secondary logic can control the local pulse generator to generate a biphasic pulse at the specified amplitude. During the biphasic pulse, the primary logic switches off SW1 and SW2 to isolate the Secondary #1 ASIC from the other stimulators. This isolation prevents a potential current path from the stimulating electrodes to electrodes connected to other stimulators and minimizes inter-channel electrical crosstalk. During isolation, the secondary ASIC is supplied by the energy storage capacitor, C1. At the completion of the biphasic pulse, SW1 and SW2 are turned on again to recharge C1 until the next biphasic pulse. SW1 and SW2 are implemented in the primary ASIC with complementary HV MOSFETs. When Secondary #1 ASIC is isolated, the voltage at the negative terminal of C1, VSS_S1, may be lower than the substrate voltage of the primary ASIC at VSS. To ensure the NMOS in SW2 remains off, a diode D2 is added in series with SW2. Similarly, the voltage at the positive terminal of C1, VDDH_S1, may be higher than VDDH, which is the bias voltage of the NWELL for the PMOS in SW1. Diode D1 in series with SW1 ensures the PMOS in SW1 remains off. The storage capacitors C1 and C5 are 2.2 μF. When supplying a biphasic pulse at 3 mA with an overall pulse width of 1 ms, the voltage drop across the storage capacitor is (3 mA × 1 ms)/2.2 μF = 1.36 V. The on resistance of SW1, SW2, SW3, and SW4 is 28 Ω each, and the RC time constant when recharging the storage capacitors is 123.3 μs. For the maximum specified pulse frequency is 500 pulse per second (pps), this time constant is sufficiently fast for recharging the storage capacitors during the pulse interval.

Figure 2E shows the circuits of the pulse generator in Stimulator Secondary #1. The pulse generator in each ASIC consists of an 8-bit binary-weighted current digital-to-analog converter (DAC) with a resolution of 1 μA, an output stage with active feedback to amplify the DAC current by four times, and a “H-bridge” formed by S1–S4 to shape the current into a stimulus biphasic pulse. The width of the cathodic phase and anodic phase are programmable so that the biphasic pulse can be either symmetrical or asymmetrical with a longer anodic phase at a lower current amplitude. This is specified by the stimulation settings, which can be programmed from the remote host computer. The anode node between S1 and S3 connects via a 1 μF blocking capacitor (C_b1_) to a 1-to-6 multiplexer, and the cathode node between S2 and S4 to a 1-to-2 multiplexer, also via a 1 μF blocking capacitor (C_b2_); thus, the stimulation is selectable between 1 of 6 stimulating electrodes and 1 of 2 return electrodes. In each secondary stimulator a 1:3 current booster outside the ASIC further increases the maximum stimulating current to 3 mA. As shown in Figure 2E the current booster is implemented as a source-degenerated current mirror using a discrete matched transistor array (THAT380 IC). Between current pulses, the pulse generator is connected to VDDH and VSS but switches S1, S2, S_c_, and S5 are off to isolate the electrodes from VDDH and VSS, preventing stimulating current from other stimulators from flowing into these electrodes. S3 and S4 stay on during the pulse interval for removing any remaining charges on the electrodes due to mismatch or charge leakage.

### Power and Data Telemetry

**FIGURE 3:**
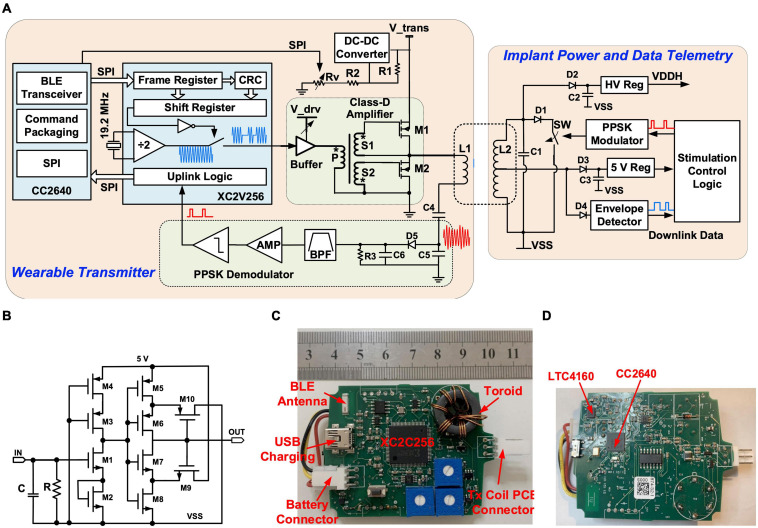
Stimulator circuits: **(A)** Schematic of the power and data telemetry; **(B)** Schematic of the integrated OOK demodulator; **(C)** Photo of the top side of the wearable transmitter; **(D)** Photo of the bottom side of the wearable transmitter.

The implantable stimulator is powered by and communicates to the wearable transmitter via a power and data telemetry over an inductive link (Donaldson and Perkins, 1983; Schormans et al., 2018). The circuit arrangement of the power and data telemetry is shown in Figure 3A. The inductive link comprises a 5-turn, 32-mm diameter Tx coil and a 7-turn, 28-mm diameter Rx coil. Both coils are solenoids wound using 0.5 mm gauge enameled copper magnet wires. The Tx coil, *L1*, is driven by a class-D amplifier consisting of two NMOS transistors, M1 and M2, using discrete IRLML2030 N-channel power MOSFETs. M1 and M2 are switches turning on and off at 9.6 MHz. Their gates are driven by the secondary sides, S1 and S2, of a toroidal transformer, in opposite polarity. The toroidal transformer arrangement ensures a non-overlap time between the switching on of M1 and M2 to avoid shoot through current. The primary side of the toroidal transformer, P, is driven by a Xilinx XC2V256 complex programmable logic device (CPLD) through a buffer 74AC14. The supply voltage of the class-D amplifier is provided by a dc-dc converter LT1615 with a programmable feedback resistor implemented with a potentiometer AD5220; thus, the transferred power over the inductive link can be controlled by the MCU. The Rx coil, L2, is tuned at 9.6 MHz with capacitor C1. The voltage across coil L2 is rectified by the Schottky diode D2 and a 10 μF capacitor C2, and regulated by a high voltage regulator (MIC5233) to a stable dc supply voltage VDDH of 16 V. There is also a middle tap on L2, where the voltage is rectified by D3 and C3 and then is regulated by a 5 V linear regulator in the primary ASIC to a 5 V supply for the low voltage circuits in the implant. The wearable transmitter is supplied by a lithium polymer battery LP-443440 (3.7 V, 560 mAh). The battery can be recharged from a USB port, regulated by a power management IC (LTC4160).

The inductive link also functions as a bidirectional half-duplex communication channel. The downlink data stream consisting of control commands and stimulation parameters are sent to the implant using on-off keying (OOK) modulation. The uplink data from the implant are transmitted using passive phase-shifted keying (PPSK) modulation (Jiang et al., 2017). During downlink data transfer, the MCU on the wearable transmitter sends stimulation settings received from the host computer over the BLE radio to the CPLD via a serial peripheral interface (SPI). The data frames are shifted in series at 400 kb/s to control an internal switch to turn on and off a 9.6 MHz output signal, which drives the class-D amplifier. On the Rx side, the data stream is recovered from the modulated carrier at the middle tap of L2, where the carrier is first rectified by D4, and then the envelope is extracted by an integrated envelope detector (ENV) and a Schmitt trigger in the primary ASIC. The circuit of the integrated OOK demodulator is shown in Figure 3B. The uplink data transfer is implemented with an integrated PPSK modulator. A logic “1” transmitted shorts L2 using an integrated switch SW when the voltage across L2 crosses zero from the negative value and holds for 1.5 carrier cycles. As a result, a current surge is caused in L1 and causes a transient voltage peak on the tuning capacitors C4 and C5. The transient voltage on C5 is demodulated through a passive envelope detector formed by D5, R3, and C6, and then is filtered and amplified. The bitstream is then extracted using a comparator. Details of the PPSK demodulation circuits are presented in Jiang et al. (2017). L1, C4, C5, D5, R3, and C6 are mounted on the Tx coil PCB and the rest of the transmitter circuitry is located inside a wearable unit, as shown in the photos in Figures 3C,D. In the implant, L2, C1–C3, and D1–D4 are located on the Rx coil PCB, and the rest of the implant electronics are mounted on the hybrid which is hermetically sealed with a ceramic lid.

### Implant Packaging and Encapsulation

**FIGURE 4:**
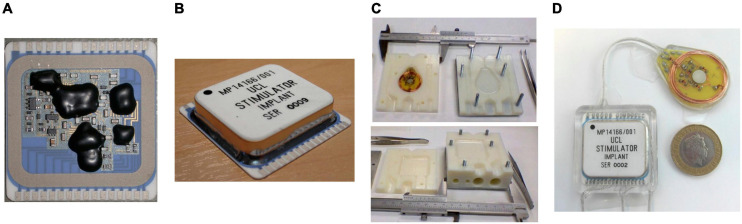
Implant fabrication and encapsulation: **(A)** Stimulator circuits assembled on a ceramic substrate with the bare dies covered in epoxy glob top; **(B)** Stimulation circuits hermetically sealed in a metallized ceramic lid; **(C)** 3D-printed mold for encapsulation: mold for the hybrid (top) and mold for the Rx coil PCB (bottom); **(D)** fully encapsulated implant with a £2 coin as reference for the size.

The implantable hybrid was constructed from a 36 mm × 38 mm ceramic substrate (96% alumina) with a thickness of 0.635 mm. Two layers of tracks were screen printed in thick film gold (8844-G Au) of 8 ± 1 μm thickness, with solder pads in the top layer over-printed with thick film platinum-gold (5837-G PtAu) of 12 ± 2 μm thickness. Thick film multilayer dielectric (4913-G) was over-printed between the layers and also on top of the top layer, covering all tracks except for the solder pads. A seal ring was formed by screen printing a platinum-gold layer; this also created hermetic feedthroughs for dielectric covered tracks from the hybrid circuit. All discrete components were soldered to the hybrid and flux residue was cleaned by sequential washes in acetone, propan-2-ol (isopropanol), and de-ionized water with ultrasonication. The primary stimulator ASIC, two secondary ASICs and two THAT380 bare dies were wire-bonded to the substrate, as shown in Figure 2B, and covered with epoxy glob top (Ablestik 968-2), as shown in Figure 4A. The hybrid was dried and sealed hermetically using a custom-made metallized ceramic lid (A473, Kyocera) of 32.13 mm × 28.55 mm size, 6.2 ± 0.5 mm height, and 3 mm thickness, soldered to the screen-printed seal ring while the assembly was placed on a hot plate at 150°C. Figure 4B shows the hermetically sealed hybrid.

The circular Rx coil PCB was constructed on an FR4 printed circuit board with a diameter of 34 mm, onto which were mounted discrete passive components in individual hermetic packages, the alignment magnet, and a coil of 0.5 mm gauge enameled copper magnet wire. The PCB was constructed without solder resist and silkscreen, and with exposed copper traces (no pad finish) to improve encapsulant adhesion.

The hybrid and Rx coil PCB were joined with multistrand fluoropolymer insulated stainless steel Cooner Wire (AS632, Cooner Wire Company, Chatsworth, CA, United States), which was also used for electrode connection cables. Connection wires were threaded through 1 mm bore silicone rubber tubes and soldered to form implantable cables. Three electrode connection cables were formed, one for each parallel stimulator ASIC with the associated two stimulation and six return lines. Electrode cables were terminated with miniature connectors (Nano 360^®^ Plastic Circulars, NCS-11-DD, Omnetics Connector Corporation, Minneapolis, MN, United States).

The hermetically sealed, soldered hybrid and the Rx coil PCB were cleaned by sequential washes in acetone, propan-2-ol, de-ionized water, Leslie’s soup, and de-ionized water, with each cleaning stage ultrasound assisted. Leslie’s soup is a mixture of 0.5 wt% detergent (Teepol-L, Teepol Products, Kent, United Kingdom), and 25 wt% trisodium phosphate (anhydrous, 13438, Alfa Aesar, Heysham, United Kingdom), in de-ionized water. Cleanliness before encapsulation is essential for the survival of long-term implants (Vanhoestenberghe and Donaldson, 2013; Lonys et al., 2017; Kiele et al., 2020). Following rinsing, the conductivity of the rinse solution was monitored to confirm adequate cleanliness.

The cleaned implants are encapsulated in silicone rubber. A low viscosity, two-part silicone adhesive (EPM-2420, Avantor-NuSil, Radnor, PA, United States) was used to reduce the risk of voids and bubbles. EPM-2420 is mixed in a 1:1, Part A: Part B ratio using a speed mixer for 2 min at 2500 rpm (Dual Asymmetric Centrifugal Laboratory Mixer System, DAC 150 FVZ-K, Synergy Devices Ltd). Two molds were designed in Autodesk Inventor Fusion 2013 for the hybrid and Rx coil PCB. The molds were 3D printed in Verowhite Plus plastic with its gloss finish, to 0.1 mm precision, as shown in Figure 4C. Companion “Dural” plates were machined so the mold halves could be bolted together. Implants were held in the cleaned molds using pre-formed silicone spacers. Implants were encapsulated under vacuum (60 mBar) in a centrifuge (up to 200 *g*) to remove air bubbles. Because of the 65°C temperature limit of the Verowhite mold, the silicone rubber was cured at 60°C for 4 h. The implants were extracted from the mold, and sections of Dacron reinforced silicone rubber sheet were glued to the encapsulated implant with EPM-2420 to create suture sites for surgery. Free from the molds, the silicone rubber was further cured for 1 h at 80°C to complete the encapsulation. The encapsulated implant is shown in Figure 4D. An alternative medical grade silicone (MED-6215, Avantor-NuSil) is under investigation for long-term implantable devices.

Cleaned miniature connectors are also encapsulated in silicone rubber (Lancashire et al., 2021). Silicone tubing was placed at the base of each connector, surrounding the soldered wire ends. EPM-2420 silicone was degassed at 30 mbar in a vacuum centrifuge for between 1 and 3 min, until bubbles were no longer visible, nor flew onto the wire ends, covering all exposed solder. Silicone was cured at 80°C for 3 h under pressure (2.5 bar) to shrink any bubbles present.

### Stimulation Control Procedure

#### Graphical User Interface

**FIGURE 5:**
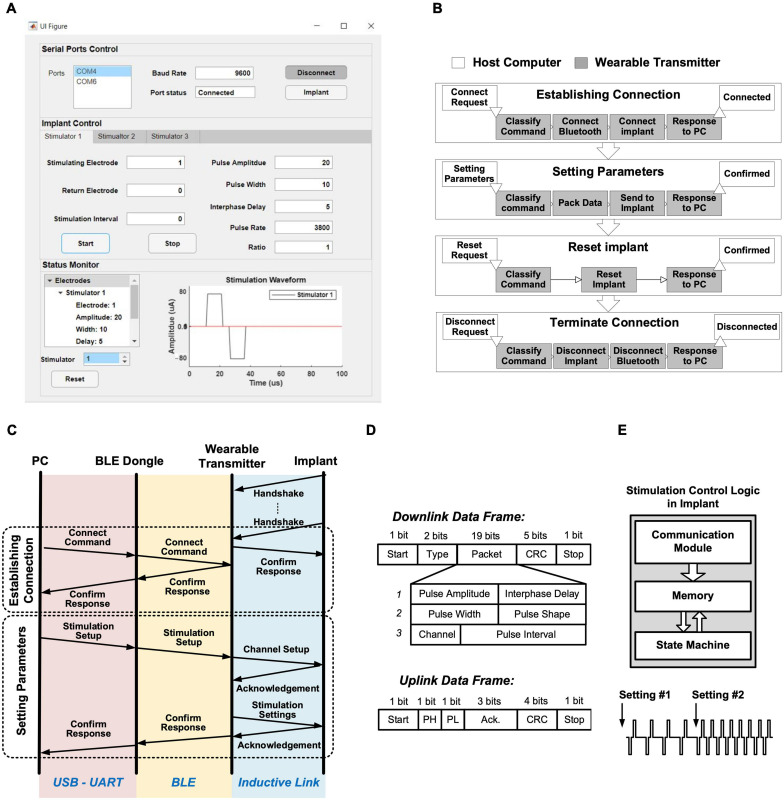
Stimulation control protocol: **(A)** Graphical user interface; **(B)** Four procedures for operating the implant from a host computer, with flowcharts on the wearable transmitter in each procedure; **(C)** Overall communication procedure between the host computer and the implant for establishing connection and setting stimulation parameters; **(D)** Structure of the data frames over the inductive link; **(E)** Block diagram of the stimulation control logic in the implant, with an example of changing stimulation parameters on-the-fly.

A GUI was developed in Matlab R2020a (MathWorks, Natick, MA, United States) for remotely controlling the stimulation on-the-fly. The GUI controls the Bluetooth connection, implant connection, and stimulation parameter settings. The layout of the GUI is shown in Figure 5A. The top panel “Serial Ports Control” controls the Bluetooth and implant connect/disconnect functions, including selecting the COM port number and setting the baud rate. The default baud rate is 9600 b/s. The middle panel “Implant Control” sets the stimulation parameters, where the three identical setting tags, “Stimulator 1,” “Stimulator 2,” and “Stimulator 3,” are provided for each stimulator ASIC. The setting parameters for each stimulator ASIC include selection of the stimulating and return electrodes, the amplitude and width of the biphasic current pulses, the delay between the cathodic and anodic phases, the pulse rate, the shape of charge-balanced biphasic pulses (symmetrical or asymmetrical), and the length of a pulse train. The lower panel “Status Monitor” displays the received stimulation parameters and the expected waveform of current pulses. In the example shown in Figure 5A, the setting parameters specify stimulation between electrodes E1 and E8 on Stimulator ASIC Primary, with symmetrical biphasic current pulses with 80 μA (setting step size 4 μA) in amplitude, 10 μs width (setting step size 1 μs) and a pulse period of 1 ms [after reverse exponential conversion (Jiang et al., 2011)]. The expected waveform is shown in the Status Monitor.

#### Control Procedure

The backend software communicates with the wearable transmitter via a BLE radio link, relayed by the BLE dongle. The communication facilitates four different procedures: establishing connection, sending stimulation parameters, reset implant, and terminating connection, as shown in Figure 5B (the operations in the light boxes are executed by the host computer and those in the shaded boxes by the MCU module CC2640 on the wearable transmitter).

The overall communication procedure between the GUI and the implant for controlling multi-channel stimulation is shown in Figure 5C. During the “establishing connection” procedure, the link between the implant and the wearable transmitter, and the link between the wearable transmitter and the PC, are established separately. After the implant is powered, it sends a handshake request to the wearable transmitter via the inductive link every 250 μs until it receives confirmation from the transmitter, after which communication over the inductive link is established. The communication between the PC and the wearable transmitter is established after a “connecting” request is sent from the GUI with a specified baud rate and serial port number, where the host computer then wakes up the BLE dongle to establish Bluetooth connection with the wearable transmitter. The MCU on the wearable transmitter classifies the received command and sends a confirmation back to the host computer, hence the communication between the host computer and the implant is established, as shown in Figure 5B.

Stimulation from the implant can be controlled from the PC following the “setting parameters” procedure, where stimulation parameters set in the GUI are sent in packets via the Bluetooth link to the transmitter, which repackages the data into frames shown in Figure 5D and forward the frames to the implant over the inductive link. The implant verifies the received setting parameters using cyclic redundancy check (CRC) in the frames, and sends back an acknowledgment frame to the transmitter with indicating whether the parameters are correctly received or a resend is needed. When all parameters are correctly received, the wearable transmitter sends confirmation back to the host computer to complete the “setting parameters” procedure. This procedure is repeated when changes to the stimulation are needed. After each procedure, the stimulation control logic in the primary stimulator ASIC stores the settings in a built-in memory, where the state-machine for the selected stimulator repeatedly loads the parameters from the memory for continuously generating stimulation pulses as specified, as illustrated in Figure 5E (Jiang et al., 2011). Figure 5E illustrates the change in stimulation pulses by the two “setting parameters” procedures.

The operation of the implant can be reset by the “reset implant” procedure. When reset is requested from the GUI to the transmitter, as shown in Figure 5B, the transmitter will temporarily terminate the power delivery to the implant, which forces the implant to conduct a power-on reset. This safety feature provides an emergency exit to terminate stimulation from the implant during experiments. After an experiment session, the Bluetooth link between the PC and the transmitter can be released by the “terminate connection” procedure, as shown in Figure 5B.

## Results

The performance of the fully implantable stimulator was evaluated by electrical and *in vitro* experiments with electrodes in saline, and by accelerated lifetime testing.

### Feasibility for Chronic Implantation

The quality of the seal of the implantable hybrid was tested according to the MIL-STD-883 (MIL-STD-883L, 2019) standard test for hermeticity. After sealing the lid, the package was bombed in helium for 2 h at 2 bars, then transferred (maximum delay 1 h) to a mass spectrometer for a fine leak test. The test was considered passed if the leak rate was lower than 5 × 10^–8^ atm cc/sec helium. After passing this fine leak test, the hybrid was placed in gross leak tank at 125°C for 1 min, and the package was considered to be sufficiently hermetic if no bubbles were observed. Following this procedure, for hybrids that pass the tests, the minimum time for the internal humidity was estimated to reach 60% RH to be at least 151 days, or 47 days minimum to reach 30% RH. Note that the actual times are likely to be much longer because the actual leak rate is likely to be much lower, but the exact leak rates is not available as the MIL-STD-883 standard only specifies a pass/fail fine leak test (Vanhoestenberghe and Donaldson, 2011).

**FIGURE 6:**
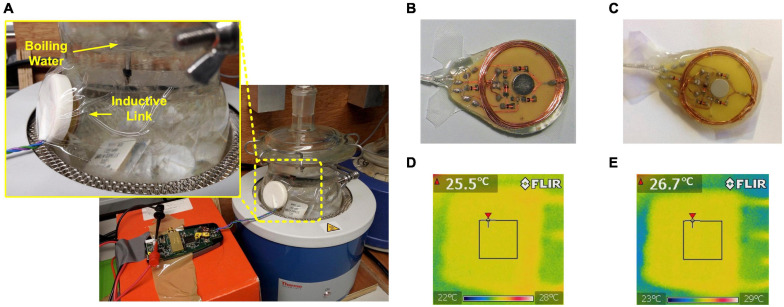
Evaluation of feasibility for chronic implantation. **(A)** Setup of accelerated lifetime test; **(B)** Photo of the Rx coil PCB before the accelerated lifetime test; **(C)** Photo of the Rx coil PCB after accelerated lifetime test for 15 days; **(D)** Thermal image of the implant immediately after switched on at room temperature; **(E)** Thermal image of the implant 1 h after switched on in room temperature.

The suitability of the implantable stimulator for chronic implantation was evaluated by accelerated lifetime testing. The test setup is shown in Figure 6A. The implant was placed inside a round bottom flask filled with de-ionized water. Deionized water was used to reduce the challenge of evaporation changing saline concentration. The silicone rubber used has low permeability to metal salts (Donaldson et al., 2011) and high permeability to water vapor, the most likely failure mode is driven by the penetration of moisture through the encapsulation layer (Donaldson, 1996). Should there be any ionic contamination on the implant surface (underneath the silicone encapsulation layer), then the osmotic gradient driving water molecules toward the contaminant and contributing to forming a pocket of liquid water, is worse in deionized water than in saline, further accelerating the failure rate. Therefore, for an implant of this type fully encapsulated in silicone, long-term tests in deionized water are appropriate. The flask was continuously heated on a heating mantle (Thermo Fisher Scientific, Waltham, MA, United States) at the boiling temperature for 15 consecutive days. A reflux condenser was installed on the flask to keep the volume of the de-ionized water unchanged. The implant was inductively powered by the first version of the wearable transmitter (Jiang et al., 2016) continuously during the entire course of the accelerated lifetime test. Data packets from the implant were frequently checked to monitor the status of the implant electronics and to ensure the wireless power transfer at a level sufficient for operation. Figure 6B shows the Rx coil PCB before the accelerated lifetime test, and Figure 6C shows the Rx coil PCB immediately after the implant was extracted from the flask at the end of the 15-day test. No corrosion was observed.

After returning to room temperature, the implant was placed in de-ionized water at room temperature but with the top surface exposed to open air. The implant was inductively coupled with the wearable transmitter and the three stimulators were set to generate current pulses at the maximum amplitude at 500 pps. Immediately after the stimulation starts, a thermal image was taken using a FLIR E4 thermal imaging camera (FLIR Systems, Wilsonville, OR, United States). The surface temperature was 25.5°C, as shown in Figure 6D. The implant was allowed to continuously operate for 1 h at the same settings, then the surface temperature was measured again, which increased to 26.7°C, as shown in Figure 6E.

### Electrical Performance Evaluation

**FIGURE 7:**
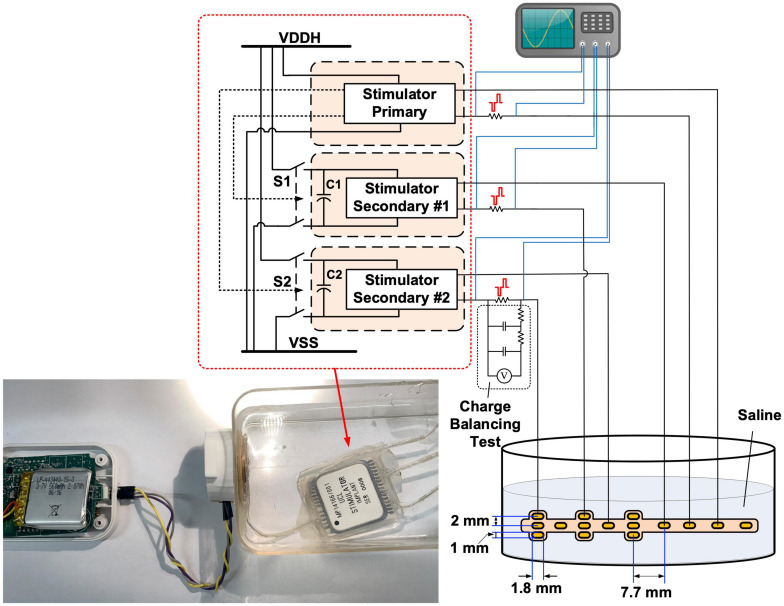
Diagram of the *in vitro* benchtop performance evaluation setup. Insert: photo of the wearable transmitter and implantable stimulator during the in vitro experiment.

The electrical performance of the implantable stimulator was evaluated with *in vitro* experiments both before and after the accelerated lifetime test. No changes were observed. Figure 7 illustrates the setup of the *in vitro* experiments, where the implant was submerged in saline solution (16.7 mS/cm^2^) and was inductively coupled with the wearable transmitter. An epidural electrode array (Courtesy of Fraunhofer IMM) (Capogrosso et al., 2016) was connected to the implant and was also submerged in saline solution. There are 15 gold electrodes in a size of 1.8 mm × 1 mm on a polyimide substrate distributed to the three stimulators. Sensing resistors were connected in series with the electrode array for measuring the current pulses using a DSO-X 2024A oscilloscope (Keysight, Santa Rosa, CA, United States). Stimulation was set from the GUI on a remote host computer. Note that the epidural electrode array was not included in the accelerated lifetime test.

**FIGURE 8:**
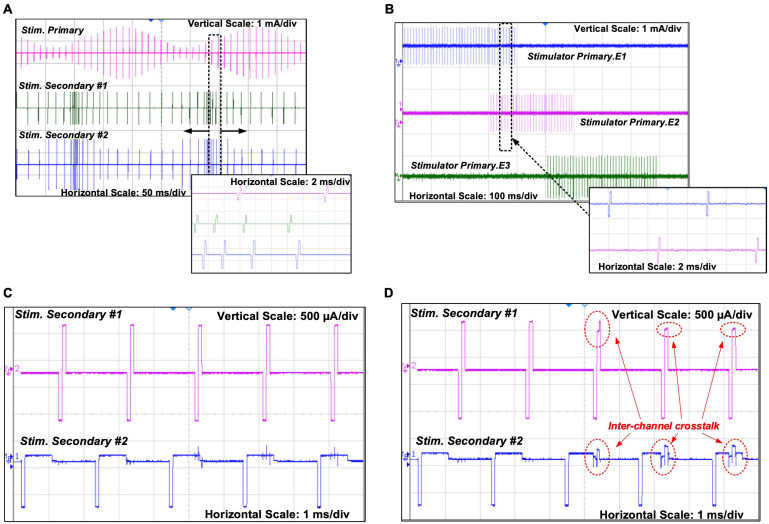
*In vitro* electrical performance evaluation: **(A)** Parallel stimulation on the three stimulators in different modes of modulation; **(B)** Pulses multiplexed among electrodes on the primary stimulator; **(C)** Concurrent stimulating pulses from the two secondary stimulators with minimized crosstalk; **(D)** Concurrent stimulating pulses from a benchtop setup with the same circuits but without the power-isolation scheme.

**FIGURE 9:**
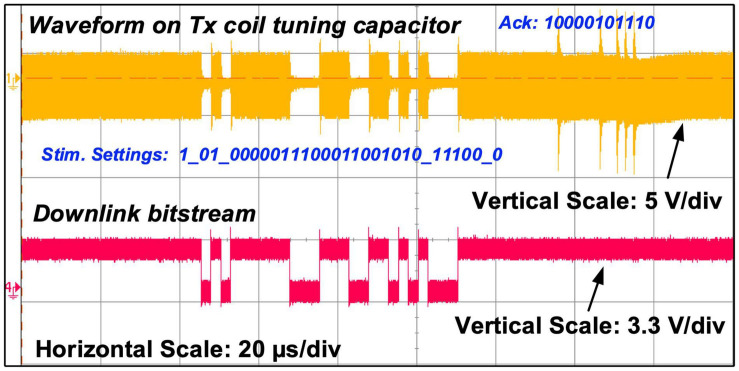
Measured carrier waveform on the Tx tuning capacitor showing both OOK and PPSK modulation, alongside the measured downlink bitstream.

The results shown in Figures 8A–C, 9 were recorded after the accelerated lifetime test. Figure 8A shows parallel stimulation from the three stimulators. The modulation patterns in this test were prestored in the CPLD on the wearable transmitter, where the remote host selected the patterns by their identification number. All stimulators were set to generate symmetrical biphasic pulses with a pulse width of 200 μs. Pulses on the primary stimulator were at a constant pulse rate of 100 pps but the amplitude was modulated sinusoidally at 4.5 Hz between 250 μA and 1 mA. Pulses on Stimulator Secondary #1 were at a constant amplitude of 800 μA but the pulse rate was modulated sinusoidally at 4.5 Hz between 400 pps and 53 pps. Pulses on Stimulator Secondary #2 were modulated both in frequency and amplitude, where the frequency modulation was the same as that on Stimulator Secondary #1, and the amplitude modulation was a 2-level step change between 700 μA and 1 mA, also at 4.5 Hz. Figure 8B demonstrates multiplexing stimulation among electrode pairs on the primary stimulator. In the first 300 ms, stimulation was from electrodes E1 and E8, with a current amplitude of 800 μA at a pulse rate of 100 pps. From 300 ms to 400 ms, the pulse rate was increased to 200 pps, and the pulses were multiplexed between E1 and E2 at a fixed interval of 5 ms, as highlighted in the zoom-in view, effectively providing 100 pps stimulation at both E1 and E2. After 400 ms, the pulse rate was changed back to 100 pps and stimulation was from E2 only. This process repeated after 500 ms between E2 and E3. The tests shown in Figures 8A,B were repeated for all the 36 electrode pairs.

Figure 8C shows the two secondary stimulators delivering concurrent biphasic pulses to the electrode array in saline solution (16.7 mS/cm^2^). The pulses from Stimulator Secondary #1 are symmetrical, and the pulses from Stimulator Secondary #2 have anodic phases 8 times longer in width than the cathodic phases, and 8 times lower in amplitude to retain charge balance. Minimal spikes can be seen on the pulses from Stimulator Secondary #2 when they occur at the same time as the pulses from Stimulator Secondary #1. To compare the crosstalk reduction performance, a benchtop stimulator using the same stimulator ASICs in the same circuit arrangement was tested in the same setup, but the two secondary stimulator ASICs are constantly connected to the power rails without isolation. The measured pulses are shown in Figure 8D. Significant distortion can be observed on pulses from both stimulators when they occur at the same time. As shown in Figure 2E, the stimulating current between an electrode pair is generated from the current source and passes through the electrode pair via the H-bridge formed by S1 – S4 toward VSS_S1. When current pulses on both the stimulators occur at the same time, if VDDH_S1 and VDDH_S2, as well as VSS_S1 and VSS_S2, are shorted, part of the stimulating current on Stimulator Secondary #2 finds a pathway to VSS_S1 via the stimulating electrode pair on Stimulator Secondary #1, resulting in the distortion on the current pulses, as shown on the last two pulses in Figure 8D which are measured on the stimulators on the current path from the electrode pair to VSS_S1 (or VSS_S2), as shown in Figure 7. The first two biphasic pulses on Stimulator Secondary #2 in Figure 8D occur in the pulse interval on Stimulator Secondary #1, while S1, S2, and S5 in Stimulator Secondary #1 are off, as the crosstalk current pathway does not exist and the measured pulses are intact.

The charge balancing performance of the stimulators was evaluated using the test setup shown in Figure 7, where the dc voltage across the sensing resistor was measured after a second-order low-pass filter with a cut-off frequency of 0.28 Hz. The stimulator under test was set to generate biphasic pulses at 500 pps, with a current amplitude of 1 mA and a pulse width of 100 μs per phase. The stimulation operated continuously for 8 h, and the dc voltage across a 790 Ω sensing resistor was measured every 30 min. The measured voltage remained constant at ∼ 1 μV, suggesting a residue dc current of ∼ 1.27 nA. There are various safety limits for neurostimulation reported in literature on the residue dc current, for example, 25 nA (Sit and Sarpeshkar, 2007) and 100 nA (Huang et al., 1999). The actual safety threshold should be placed in context with the equivalent charge density and the location of the electrodes. Nevertheless, the measured dc current is much lower than these safety limits.

Figure 9 shows the measured modulation on the carrier over the inductive link for sending bitstreams in both directions. The upper waveform is the voltage measured at the input of the envelope detector in the Tx coil PCB, i.e., the voltage across capacitor *C5* in Figure 3A, and the lower waveform is the control signal for the OOK modulation switch in the CPLD. It shows a carrier modulated first in OOK to send a 28-bit stimulation setting frame to the implant at 400 kb/s, and then is modulated in PPSK by the implant to send back a 11-bit acknowledgment frame at 600 kb/s.

A battery life test showed the voltage of a fully charged lithium polymer battery (LP-443440, 3.7 V, 600 mAh) dropped from 3.7 V to 3 V after 4 h continuous operation powering and controlling the implantable stimulator.

## Discussion and Conclusion

The design, implementation and testing of a wireless fully implantable multichannel neural stimulator was described. The features and performance of the stimulator are summarized in Table 1.

**TABLE 1:** Features and performance of the implant system.

Hybrid dimensions	46 mm × 42.8 mm × 8.8 mm
Weight	35.2 g
Packaging	Hermetically sealed ceramic package with silicone encapsulation
Stimulator ASICs	0.6-μm HV CMOS
Supply voltage	5 V (digital circuits), 16 V (stimulator output stage)
Implant supply	Wireless inductive powering
Wearable transmitter supply	Rechargeable 3.7 V battery
Power consumption (stimulators)	31 mW*
Stimulation type	Biphasic constant current pulsatile stimulation
Stimulation amplitude	Primary ASIC: 8-bit current DAC, 0 μA – 1 mA, step size 4 μA Secondary ASICs: 8-bit current DAC, 0 μA – 3 mA, step size 12 μA
Pulse rate	1–500 pps, resolution ≤0.5 pps
Pulse duration	Cathodic phase: 0–500 μs Anodic phase: 1–8 times cathodic width
Number of stimulators	3, each driving 6 stimulating electrodes and 2 return electrodes
Number of electrode configurations	36
Inductive link parameters	*Primary coil*: 32 mm diameter, 0.5 mm gauge, 5 turns, 1.85 μH *Secondary coil*: 28 mm diameter, 0.5 mm gauge, 7 turns, 2.52 μH
Inductive link working distance	3–11 mm
Received DC supplied voltage	<17 V on the full receiver coil
Inductive link data rate	9.6 MHz carrier frequency, 400 kb/s OOK downlink, 600 kb/s PPSK uplink
Remote control radio link	Bluetooth Low Energy
Control interface	Matlab-based GUI for real-time stimulation control
Stimulator response latency	9.4 ms

*Measured with the three stimulators all generating biphasic pulses at 40 pps with 1 mA in pulses amplitude and 100 μs per phase.

The goal of this research was to develop a fully implantable device capable of multisite neural stimulation suitable for chronic studies in free moving animals, where the stimulation can be precisely delivered to the target sites and can be modified wirelessly in real-time from a remote-control host. The feasibility of the implantable stimulator for implantable operation and chronic implantation was evaluated. The device surface temperature rise and the external wearable device battery life for continuous operation were examined. According to BS EN 45502-1:2015 (BSI, 2015), the temperature rise caused by heat dissipation of an implantable device should be lower than 2°C. The tests conducted in this study show a 1.2°C surface temperature rise after 1 h continuous operation at the maximum stimulation capacity, which satisfies the safety criterion. The battery in the wearable device can support the system continuously working for 4 h before the battery voltage drops by 0.7 V to 3 V; 4 h is sufficient for one session of an animal experiment. The 4-h time window could be extended by increasing the supply voltage of the class-D amplifier to increase the reduced power transfer level.

To examine the feasibility of chronic implantation, the implant was evaluated in accelerated lifetime testing at 100°C for 15 consecutive days while powered on continuously. To estimate the equivalent lifetime at body temperature, instead of the empirical “10-degree rule” (Leenson, 1999), the Arrhenius equation (Zhou and Greenbaum, 2010) was used. Activation energies are rarely published, and none for hybrid circuits on alumina were found, but similar work suggests it may range from 0.7 eV to 0.4 eV (Vanhoestenberghe and Donaldson, 2013; Lonys et al., 2015). Using the lowest published activation energy of 0.4 eV, the acceleration factor is 18, or about 270 days at body temperature. For activation energy of 0.7 eV, the acceleration factor is roughly 84, suggesting an equivalent lifetime of ∼ 1,260 days at body temperature, which is sufficient for most chronic animal studies with active implantable devices reported in the literature. The ceramic packaging approach has the advantage of hermetic sealing over polymer packaging methods (Dahan et al., 2012; Au et al., 2020), and is cheaper than approaches using metallic protection.

The electrical performance evaluation demonstrates the stimulator’s capability of providing versatile stimulation on multiple channels under real-time remote control. More importantly, it demonstrated that the implantable stimulator can provide truly parallel multichannel stimulation where current pulses on different channels can occur at the same time with minimal channel interference due to crosstalk. This feature allows simultaneous, highly selective stimulation to multiple sites, especially with different stimulation patterns on each site.

The implantable stimulator provides a versatile platform for chronic experimental studies with freely moving animals for applications involving peripheral nerves, such as vagus nerve stimulation, spinal cord injury and hand neural prostheses. Stimulation for up to 36 different electrode pairs can be facilitated and connections to the electrodes are “plug & play” allowing the use of different electrodes to suit each particular application.

Future work could consider the following enhancements in the electronics:

1) *Power Consumption:* As the ASICs are implemented in 0.6 μm CMOS technology, the control logic and the low voltage analog modules such as the 8-bit current DAC are operating at a 5 V supply voltage. The power consumption of these modules is over 20 mW. Implementation in a more advanced CMOS process technology (e.g., 180 nm HV CMOS), allows the supply voltage to the control logic, biasing circuits and the current DAC to be reduced to 1.8 V or lower, reducing the power required. For example, the integrated stimulator presented in Jiang et al. (2021) is implemented 180 nm CMOS and consumes only 1 mW when generating 50 Hz, 50% duty cycle current pulses of 16 mA. The energy consumed by the output stage circuit can be reduced by dynamic compliance voltage techniques (Samiei and Hashemi, 2021) and energy recycling methods (Ha et al., 2019);
2) *Physical Size:* The power isolation scheme requires the substrate of the parallel stimulators to be separated. Using 0.6 μm CMOS technology, the stimulators have to be implemented in separate ASICs. The use of silicon-on-insulator (SOI) CMOS technology would allow a single chip solution for all the stimulator circuits in the implant. In addition, a high-frequency stimulation scheme (Jiang and Demosthenous, 2018) allows on-chip energy storage capacitors, which could further reduce the physical size of the circuit layout. Furthermore, the external current booster using THAT380 was external to the stimulator ASICs, and could be eliminated with a new stimulator ASIC design.
3) *Communication Latency:* In the first version transmitter design (Jiang et al., 2016), an easyRadio ISM module eRA900TRS was used for the communication with the remote host, chosen for its low power consumption, but it was found that the inter packet delay is not optimal. A BLE radio link is chosen for the second version transmitter design. In the 9.4 ms communication latency, 8.3 ms is from the slow UART communication between the host computer and the BLE dongle. This latency could easily be shortened by selecting a higher UART baud rate.

## Data Availability Statement

The original contributions presented in the study are included in the article/supplementary material, further inquiries can be directed to the corresponding author/s.

## Author Contributions

DJ, FL, HL, TP, and AV drafted the manuscript. DJ, FL, and MS developed the hardware and firmware. FL developed the graphical user interface. DJ, HL, TP, and AV conducted implant packaging and encapsulation. DJ, FL, MS, HL, TP, and AV designed and conducted the benchtop device evaluation. ND and AD supervised the research and reviewed the manuscript. All authors contributed to the article and approved the submitted version.

## Conflict of Interest

The authors declare that the research was conducted in the absence of any commercial or financial relationships that could be construed as a potential conflict of interest.
